# Structure boundary-preserving U-Net for prostate ultrasound image segmentation

**DOI:** 10.3389/fonc.2022.900340

**Published:** 2022-07-28

**Authors:** Hui Bi, Jiawei Sun, Yibo Jiang, Xinye Ni, Huazhong Shu

**Affiliations:** ^1^ Department of Radiation Oncology, The Affiliated Changzhou No. 2 People’s Hospital of Nanjing Medical University, Changzhou, China; ^2^ School of Computer Science and Artificial Intelligence, Changzhou University, Changzhou, China; ^3^ Key Laboratory of Computer Network and Information Integration, Southeast University, Nanjing, China; ^4^ Jiangsu Province Engineering Research Center of Medical Physics, Changzhou, China; ^5^ School of Electrical and Information Engineering, Changzhou Institute of Technology, Changzhou, China; ^6^ Laboratory of Image Science and Technology, Southeast University, Nanjing, China; ^7^ Centre de Recherche en Information Biomédicale Sino-francais, Rennes, France; ^8^ Jiangsu Provincial Joint International Research Laboratory of Medical Information Processing, Southeast University, Nanjing, China

**Keywords:** prostate ultrasound image segmentation, ASM-based key points selection, U-Net architecture, deep learning, shape prior

## Abstract

Prostate cancer diagnosis is performed under ultrasound-guided puncture for pathological cell extraction. However, determining accurate prostate location remains a challenge from two aspects: (1) prostate boundary in ultrasound images is always ambiguous; (2) the delineation of radiologists always occupies multiple pixels, leading to many disturbing points around the actual contour. We proposed a boundary structure-preserving U-Net (BSP U-Net) in this paper to achieve precise prostate contour. BSP U-Net incorporates prostate shape prior to traditional U-Net. The prior shape is built by the key point selection module, which is an active shape model-based method. Then, the module plugs into the traditional U-Net structure network to achieve prostate segmentation. The experiments were conducted on two datasets: *PH2 + ISBI 2016 challenge* and our private prostate ultrasound dataset. The results on *PH2 + ISBI 2016 challenge* achieved a Dice similarity coefficient (DSC) of 95.94% and a Jaccard coefficient (JC) of 88.58%. The results of prostate contour based on our method achieved a higher pixel accuracy of 97.05%, a mean intersection over union of 93.65%, a DSC of 92.54%, and a JC of 93.16%. The experimental results show that the proposed BSP U-Net has good performance on *PH2 + ISBI 2016 challenge* and prostate ultrasound image segmentation and outperforms other state-of-the-art methods.

## Introduction

Prostate cancer is the most common cancer among American men. The American Cancer Society estimated about 191,930 new cases of prostate cancer and about 33,330 deaths from prostate cancer in 2020 (1).

Ultrasound images can be applied for diagnosis and guide puncture and radiotherapy (2). The accurate delineation of the prostate boundary in ultrasound images is crucial for intraoperative navigation to help medical physicists operate, especially in ultrasound-guided puncture. Ultrasound-guided puncture as one of the monitoring means can reflect prostate deformation in real time.

In the era of deep learning, many convolutional neural network (CNN)-based segmentation approaches have been proposed for medical image segmentation. Fully convolutional network (FCN) is an innovative network for semantic segmentation (3). U-Net is the first CNN-based network with skip layers for biomedical image segmentation and is employed for biological microscopy images (4). 3D U-Net extends the typical U-Net architecture by replacing all 2D operations with their 3D counterparts and is applied in the volumetric segmentation of sparsely annotated volumetric images (5). U-Net++ is a new, more powerful architecture for medical image segmentation (6). The architecture of U-Net++ is a deeply supervised encoder–decoder network, where the encoder and decoder sub-networks are connected through a series of nested, dense skip pathways. The re-designed skip pathways aim at reducing the semantic gap between the feature maps of the encoder and decoder sub-networks. V-Net is employed for 3D image segmentation based on a volumetric fully convolutional neural network (FCNN) (7). The authors introduce a novel objective function based on Dice similarity coefficient (DSC). Progressive Dense V-net (PDV-Net) was employed as a 3D-CNN encoder for fast and automatic segmentation (8) to deal with situations where the number of foreground and background voxels has a strong imbalance. These U-Net architecture networks are useful for many modalities of medical image segmentation. However, they can hardly be used directly for ultrasound image segmentation because of the several defects of ultrasound imaging (e.g., attenuation, speckle, signal drop-out, low contrast, and signal shadowing).

Shape information and boundary information play a critical role in ultrasound image segmentation (9–14). Shape models bring essential information, particularly in prostate segmentation, because the anatomical structure of a healthy prostate is more likely an ellipse shape. Gong et al. (15) proposed a deformable super-ellipse model that drives shape evolution by an efficient and robust Bayesian segmentation algorithm. Badiei et al. (16) utilized image warping and ellipse fitting for prostate ultrasound segmentation. Shen et al. (17) used a statistical shape model that adopts normalized features to make prostate shape invariant to probe rotation. The experimental results of the aforementioned works showed that incorporating tissue shape information can improve segmentation accuracy. As a kind of shape model, the active shape model (ASM) can describe various shapes based on a mean position and variant modes (18). Hodge et al. extended ASM from 2D to 3D ultrasound prostate image segmentation (19). Some methods that combine ASM with other models have been proposed to improve the segmentation accuracy. Yan et al. proposed a discrete deformable model guided by partial ASM for transrectal ultrasound image segmentation (20). Bi et al. proposed a fast and accurate segmentation method using ASM with Rayleigh mixture model clustering for prostate ultrasound images (21).

Remarkably, authors started to combine shape information and deep learning. Mishra et al. proposed an FCNN with attention to boundaries conducted on the MICCAI 2011 IVUS challenge dataset resulting in a Dice index value of 0.91 (22). Chen et al. developed a new model based on deep learning, which takes into account boundary information (23). Murugesan et al. proposed a Psi-Net that joins shape and boundary into a multitask deep network that aids in ensuring the smoothness of segmentation prediction (24). Nguyen et al. proposed a consecutive deep encoder–decoder network combined with a boundary-emphasization data augmentation (25). Hou et al. proposed a robust 3D CNN with boundary correction (26). Soliman et al. proposed a novel CNN segmentation framework based on shape features described by the seventh-order Markov–Gibbs random field, which reached a high DSC of 98.37% ± 0.68% on 95 CT lung images (27). Hesse et al. improved U-Net segmentation by adding an active contour step to correct the imperfect ground-truth labels (28). Qin et al. combined superpixel and boundary information with CNNs for liver segmentation (29). Lee et al. proposed a novel image segmentation network for medical images with ambiguous boundaries (30). In summary, boundary information is helpful to improve segmentation accuracy by combining it with a neural network.

**Figure 1:**
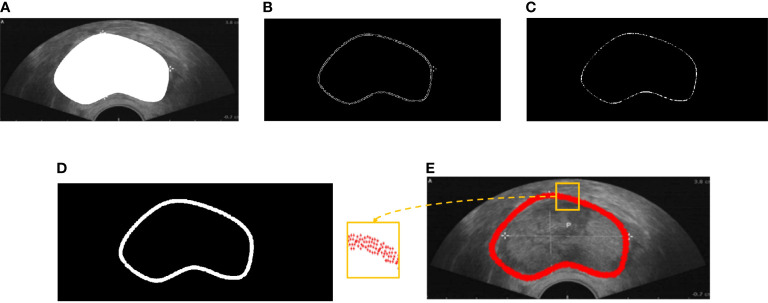
**(A)** Semantic segmentation of prostate ultrasound image. **(B)** Edge detection of the prostate. **(C)** Prostate boundary after erosion. **(D)** Erode prostate boundary after dilation. **(E)** Prostate contour.

**Figure 2:**
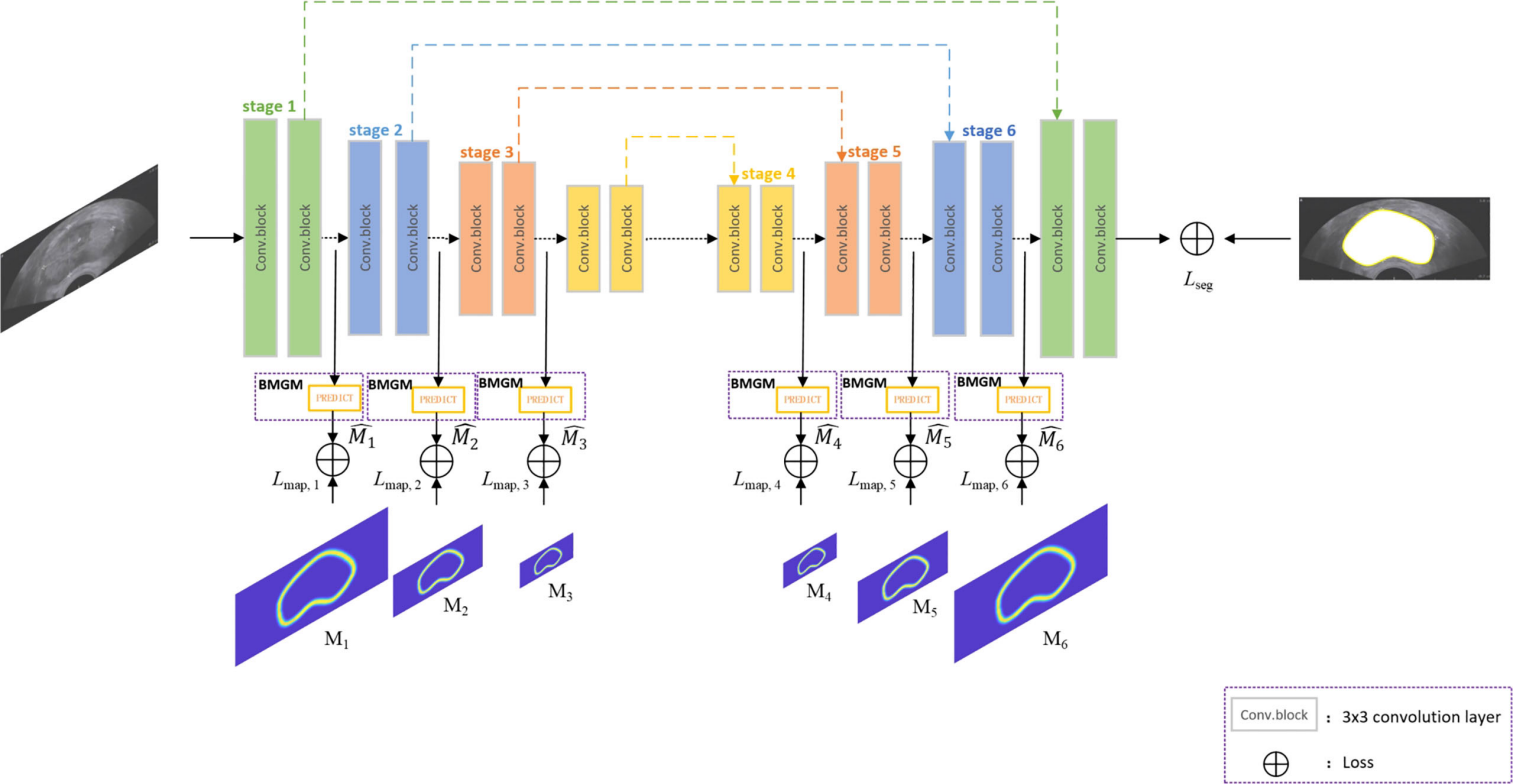
Overview of the proposed network architecture.

However, boundary representation remains a challenge in two aspects: (1) the contour of the existing dataset is implicit because the ground truth always provides semantic segmentation (**Figure 1A**), and (2) the actual contour is surrounded by many disturbing points (**Figure 1E**). In our work, we proposed a model that combines boundary information with deep learning for prostate ultrasound image segmentation. The contributions of our work are as follows: (1) we built an end-to-end neural network for prostate ultrasound segmentation; (2) an ASM-based method was utilized for boundary information extraction; (3) the key point selection models were easy to plug into any network. This paper is organized as follows. *Section 2* presents the mathematical background of ASM. *Section 3* describes the selection of the key points of initialization. *Section 4* provides the experimental results, and *Section 5* presents the concluding remarks.

## Materials and methods

### Materials

Two medical image segmentation datasets, namely, *PH2 + ISBI 2016 Skin Lesion Challenge* (31, 32) and our private dataset, were applied in the experiments. The *PH2 + ISBI 2016* dataset, which is a publicly available dataset for evaluating skin lesion segmentation, includes 900 multi-size skin lesion images and 200 dermoscopic images. The private dataset is from the Second People’s Hospital of Changzhou Affiliated with Nanjing Medical University, which was approved by the Ethics Committee of the Second People’s Hospital of Changzhou. All subjects agreed to participate in the study and signed the informed consent. All data, including 100 prostate images, are desensitization data. The ground truth was based on the average delineation from three radiologists.

### Methods

**Figure 2** shows the overview of the proposed network architecture. It consists of one encoder–decoder network and a boundary map generation module (BMGM). The BMGM was utilized to incorporate boundary information in different scales of the image within the network.

#### Boundary map generation module

**Figure 1E** shows that the points extracted from semantic segmentation have two deficiencies: (1) in each dimension, several points make key points implicit; (2) the points are chaotic. Hence, we proposed a BMGM to alleviate this situation.

First, rough prostate boundary detection was processed, including binarization, edge extraction, and morphological operation. The adaptive threshold binarization of the Otsu algorithm and the edge extraction of the Canny algorithm were adopted for ultrasound images as shown in **Figure 1B**. A morphological operation consisting of erosion and dilation was conducted to remove the disturbing points and connect the isolated points (**Figures 1C, D**). Then, the rough boundary of the prostate was detected, and its pixel points formed the rough point set.

**Figure 3:**
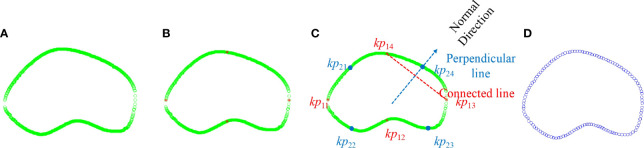
Illustration of candidate point selection and finer boundary detection. **(A)** Green—point selection after extra point removal; red—key points. **(B)** Detection of four salient points. **(C)** Interpolation of the first iteration. **(D)** Finer contour.

Second, prostate boundary finer was processed, including key point initialization and interpolation generation. Key point initialization is adopted to eliminate the disturbance of messy points. As shown in **Figure 3A**, the green circles denote only two points left on each vertical axis. Then, four salient points {*kp*_11_, *kp*_12_, *kp*_13_, *kp*_14_} were used as the initialization to demonstrate the prostate shape, where footnote 1 indicates the first iteration selection, 4 indicates the order of the points in the set (**Figure 3B**), and the red stars denote the key point set of the boundary. Then, interpolation generation was adopted to supplement the shape points to finish the finer contour detection. The interpolation needs to find the perpendicular direction of any two adjacent points. The normal direction of the perpendicular line connected to two points was achieved using Eq. (1):


(1) $$(D_{x},D_{y})=(\frac{x_{1}-x_{2}}{\sqrt{{\left(x_{1}-x_{2}\right)}^{2}+{\left(y_{1}-y_{2}\right)}^{2}}},\frac{y_{1}-y_{2}}{\sqrt{{\left(x_{1}-x_{2}\right)}^{2}+{\left(y_{1}-y_{2}\right)}^{2}}}),$$

where *D_x_* and *D_y_* are the coordinates in the *x*-axis and *y*-axis, respectively; *x*_1_, *y*_1_ are the coordinates of point 1; and *x*_2_, *y*_2_ are the coordinates of point 2. Taking *kp*_13_ and *kp*_14_ as examples, the red line denotes the connected line, whereas the blue line denotes the perpendicular line. The point was interpolated through the candidate points. Euclidean distance was used to select the candidate points so that *kp*_24_ was added to the point set as shown in **Figure 3C**. Finally, we achieved the key points as **Figure 3D** shows.

#### Network Backbone

As an attractive structure-preserving method, Lee’s model (30) selects an optimal solution from a set of random sampling on the target contour, but the extracted points are not evenly distributed. Our proposed work generates the key points over the boundary map generation module, called the BMGM, to obtain a more uniform repartition of the key points along the contour. As the replacement of the random sampling module of Lee’s model, the BMGM is proposed to provide a more accurate information on the tissue boundary in our segmentation network. The BMGM module is utilized to evaluate the intermediate segmentation at the different stages of the network after the convolutional block. The prediction at different scales is used to restrain the segmentation stage. The prediction module was constructed directly by an upsampling block.

The network is a typical U-Net architecture that has encoder and decoder parts besides skip layers as shown in **Figure 2**. The encoder part is used for semantic feature extraction, and the decoder part helps with generator segmentation. The BMGM was placed after the convolution block. In the network, the BMGM was utilized to generate the predicted map to restrain the intermediate segmentation. The BMGM was used in the encoder and decoder parts. The BMGM at different scales was used to compare the stage with the ground-truth map.

We utilized cross-entropy to evaluate the stage with the ground truth and restrain the segmentation with the true boundary in different stages. The map loss *L*_map_ is defined as:


(2) $$L_{\text{map}}=\sum_{i=1}^{n}-\left(M_{i}\text{log}\hat{M_{i}}+\left[1-M_{i}\right]\text{log}\left[1-\hat{M_{i}}\right]\right),$$

where *M_i_* is the boundary map generated by the ground truth, and $\hat{M_{i}}$ is the map achieved by the network in the *i*th stage.

We also used cross-entropy to evaluate the segmentation result with the ground truth and restrain the segmentation with the true boundary. Segment loss *L*_seg_ is defined as:


(3) $$L_{seg}=-G_{l\text{og}}S-(1-{\mathrm{G)}}_{l\text{og}}\left(1-S\right),$$

where *G* is the ground truth, and *S* is the segmentation achieved by the network.

Total loss *L*_total_ is defined as follows:


(4) $$L_{total}=L_{map}+L_{seg}.$$

### Experimental implementation

#### BMGM implementation details

**Figure 4:**
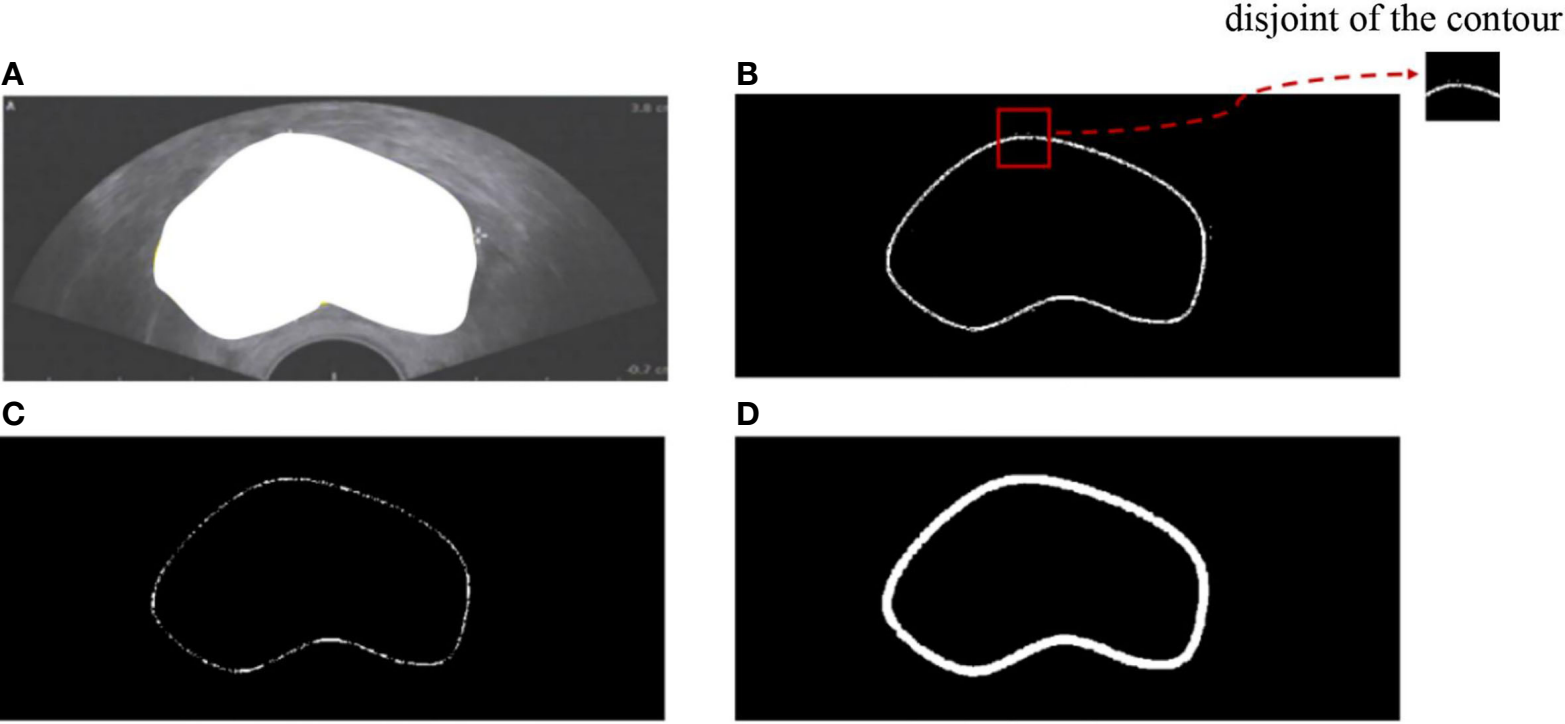
Illustration of coarse boundary detection.

In the coarse boundary step, we detected the edge of the prostate based on Otsu’s algorithm. Then, all-one template size 2×2 for image erode and disk template size 5×5 for image dilate were implemented (**Figure 4**). **Figure 4A** depicts the semantic segmentation of the prostate. The edge of the prostate can be detected through image binarization as shown in **Figure 4B**. **Figure 4B** shows that more than two points along each dimension are disturbing points. Thus, image erosion was implemented to solve this issue. **Figure 4** illustrates that the points were reduced along each dimension; however, the operation led to some disconnection along the contour. Image dilation was implemented to make contour connections as shown in **Figure 4D**. The closed contour is smooth and can be seen as the coarse contour of the prostate.

**Figure 5:**
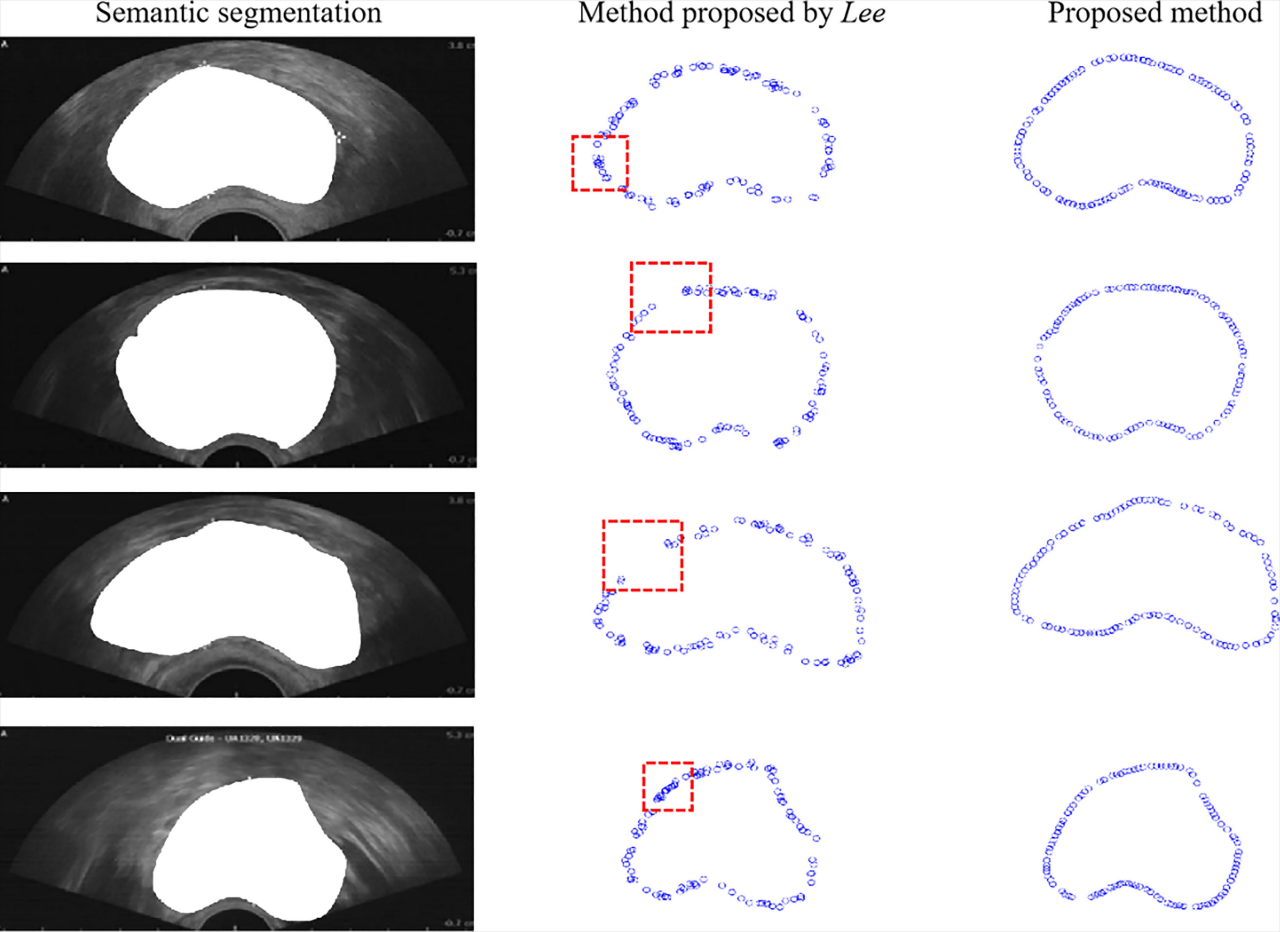
Comparison of boundary detection. The first column is the original image, the second column is the boundary detection based on Lee’s method, and the third column is the boundary detection based on the proposed method.

The proposed method was compared with the point selection method proposed in (30) to evaluate the proposed method qualitatively. We conducted experiments on 100 prostate ultrasound images. Four representative demos were used as **Figure 5** shows. The first column represents the semantic segmentation responding to each ultrasound image. The second column is the point selection based on Lee’s method (30). As the figure shows, some overlap points are present inside the red box, and disconnection occurred along the boundary. The third column shows the points selected by the proposed method. The points have an equidistant distribution along the boundary and do not overlap.

Furthermore, we used the intersection over union (IOU) to evaluate the proposed method quantitatively using Eq. (5), where *S_L_* denotes the region generated by the points based on Lee’s method and *S_P_* denotes the region generated by the points based on the proposed method. IOU equals the ratio of the overlap of *S_L_* and *S*_P_ to the union of *S_L_* and *S_P_*. We calculated the mean and variance of 100 prostate ultrasound images, and representative images are shown in **Table 1**. Most key points generated based on the proposed method showed higher IOU than those generated by Lee’s method. Furthermore, the mean (0.7280) and variance (0.0173) of the proposed method were higher than those of Lee’s method (0.7134 and 0.0191, respectively).


(5) $$\text{IOU}=\frac{S_{L}\cap S_{P}}{S_{L}\cup S_{P}}$$

**Table 1:** Intersection over union (IOU) of representative prostate ultrasound images.

Patient number/IOU	Lee’s method	BSP U-Net
#1	0.7167	**0.7316**
#2	0.6954	**0.7143**
#3	**0.7297**	0.7144
#4	0.7210	**0.7439**
#5	**0.7207**	0.7128
#6	0.7211	**0.7369**
#7	0.7198	**0.7334**
#8	0.6914	**0.7363**
#9	0.7174	**0.7325**
#10	**0.7322**	0.7087
#11	0.7249	**0.7308**
#12	0.7207	**0.7461**
#13	0.7378	**0.7404**
#14	0.7294	**0.7400**
#15	0.7206	**0.7474**
#16	0.7149	**0.7435**
#17	0.7285	**0.7362**
#18	**0.7303**	0.7293
#19	0.7140	**0.7421**
#20	0.7278	**0.7463**
*Mean*	0.7134	**0.7280**
*Std*	0.0191	**0.0173**

Bold values means best values.

#### BSP U-Net Implementation Details

All the experiments were implemented in TensorFlow 1.4.0 with an NVIDIA GeForce RTX 2080Ti GPU. We used a robust model training schema by 10-fold cross-validation and randomly shuffled all images. We trained the model by Adam optimization algorithm with an initial learning rate of 1e−4, a maximum epoch of 1,200, and a batch size of 8. The automatically saved optimal model was used to evaluate the testing set.

We evaluated the proposed method by the test set. We trained and tested other classic deep-learning based models, including U-Net (27), FCN (3), and Lee’s network (30), using the same dataset in the paper to evaluate the performance of our network objectively.

## Discussion

Structure information is crucial for medical image segmentation within a deep learning network. Derived from the capability of ASM-based methods to capture the shape variability of the prostate within an ultrasound image, we employed ASM to generate a key point map from the delineated boundary based on physicians.

Though several methods are proposed to generate shape information, the demonstration of the prostate in ultrasound images is still a challenge because of the inhomogeneous images. Lee proposed a boundary key point selection algorithm for a selected point set to demonstrate the target object. In our work, we considered the prostate boundary as an ASM model to demonstrate an inhomogeneous prostate. ASM is applied to generate the key points of a prostate boundary to achieve more homogeneous tissue information, which is helpful for ultrasound image segmentation. This assumption was verified through experiments about plugging a key point selection module based on ASM in a CNN. The key point selection module based on ASM is simple to implement and conveniently attached to a network in a plug-and-play manner.

The proposed ASM-based method outperformed Lee’s method in terms of IOU. Compared with Lee’s method, the mean intersection over union (MIOU) of the proposed ASM-based method was improved by 1.3%, and the standard deviation declined by 9.4%. The larger mean IOU means higher accuracy, and the smaller standard deviation means more stability. The reason the proposed method showed better performance is that BMGM re-partitions the key points along the prostate contour. Compared with Lee’s method, BSP U-Net had better constraints on prostate shape.

In terms of segmentation results, the proposed ASM-based method’s key point map generation plugin (traditional U-net network) achieved considerable accuracy. We conducted experiments on two datasets. PH2 data: Compared with SCDRR, the segmentation accuracy of our method improved by 11.56% for DSC and 16.55% for Jaccard coefficient (JC) as **Table 2** shown. Compared with JCLMM, the segmentation accuracy of our method improved by 15.80% for DSC. Compared with MSCA, the segmentation accuracy of our method improved by 17.62% for DSC and 22.47% for JC. Compared with SSLS, the segmentation accuracy of our method improved by 22.40% for DSC and 29.36% for JC. Compared with FCN, the segmentation accuracy of our method improved by 7.30% for DSC and 7.82% for JC. Compared with Bi, the segmentation accuracy of our method improved by 5.82% for DSC and 5.46% for JC. Compared with Lee, the segmentation accuracy of our method improved by 4.46% for DSC, and 5.08% for JC. Prostate ultrasound data: Compared with FCN, the segmentation accuracy of our method improved by 2.93% for pixel accuracy (PA), 4.84% for MIOU, 8.39% for DSC, and 5.16% for JC. Compared with U-Net, the segmentation accuracy of our method improved by 1.69% for PA, 2.95% for MIOU, 5.70% for DSC, and 3.27% for JC as **Table 3** shown. Compared with Lee, the segmentation accuracy of our method improved by 0.84% for PA, 1.42% for MIOU, 2.05% for DSC, and 1.74% for JC. The reason is that the proposed method had a better performance in homogeneous tissue presentation to achieve more accurate segmentation results.

**Table 2:** Evaluation of U-Net, FCN, Lee’s network, and the proposed network on *PH2 + ISBI 2016 challenge*.

Method	Dice Coefficient	Jaccard Coefficient
SCDRR (33)	86.00	76.00
JCLMM (34)	82.85	–
MSCA (35)	81.57	72.33
SSLS (36)	78.38	68.16
FCN (3)	89.40	82.15
Bi et al., 2017 (37)	90.66	83.99
Lee’s method (30)	91.84	84.30
BSP U-Net	**95.94**	**88.58**

Bold values means best values.

**Table 3:** Evaluation of U-Net, FCN, Lee’s network, and the proposed network on prostate ultrasound images.

Method	PA	MIOU	Dice Coefficient	Jaccard Coefficient
FCNN (3)	94.29	89.33	85.38	88.59
U-Net (4)	95.44	90.97	87.55	90.21
Lee’s Method (30)	96.24	92.34	90.68	91.57
BSP U-Net	**97.05**	**93.65**	**92.54**	**93.16**

Bold values means best values.

Furthermore, we compared the performance of using a boundary mask (**Figure 4A**) and a boundary-preserving module (**Figure 4D**). The results are shown in **Table 4**. Compared with using a boundary mask, the segmentation accuracy of using boundary sample points improved by 10.56% for PA, 11.73% for MIOU, 5.39% for DSC, and 10.42% for JC. The reason is that boundary-preserving points are more adaptive to curve changes, which represent better constraints on prostate shape.

**Table 4:** Evaluation of using boundary mask and boundary-sampled points on prostate ultrasound images.

Method	PA	MIOU	Dice Coefficient	Jaccard Coefficient
Boundary mask	87.88	83.82	87.80	84.37
Boundary sampled points	**97.05**	**93.65**	**92.54**	**93.16**

Bold values means best values.

We also conducted experiments using boundary-preserving modules in the encoder layer, decoder layer, and both layers. The results are shown in **Table 5**. The performance of boundary preservation in both layers was better than that in the encoder layer; the segmentation accuracy improved by 2.14% for PA, 3.26% for MIOU, 3.28% for DSC, and 2.08% for JC. The boundary preservation performance in the encoder layer was better than that in the decoder layer; the segmentation accuracy improved by 2.57% for PA, 1.45% for MIOU, 1.44% for DSC, and 2.63% for JC. The reason is that the decoder layer also can obtain boundary information using boundary preservation in the encoder layer. The shape constraint is augmented into the information forward transmission.

**Table 5:** Evaluation of using BSP U-Net in the encoder layer, decoder layer, and both layers on prostate ultrasound images.

Method	PA	MIOU	Dice Coefficient	Jaccard Coefficient
Boundary preserving in encoder layer	95.01	90.69	89.60	92.26
Boundary preserving in decoder layer	92.63	89.39	88.33	88.92
Boundary preserving in both encoder and decoder layers	**97.05**	**93.65**	**92.54**	**93.16**

Bold values means best values.

This work focused on tumor and gland segmentation for ultrasound images with smooth and non-convex targets. The generation of key points would drop some points when encountering the bottleneck. In the future, we will conduct further research on tissues containing a bottleneck and to make our method effective on convex and non-smooth objects. In addition, considering the relatively small dataset of ultrasound images, the 10-fold cross-validation was adopted to evaluate the proposed method. In the future, for further evaluation of the proposed method, we will collect more kinds of ultrasound images and promote the proposed method for use in other organs, such as the thyroid, uterus, and liver.

## Conclusion

BSP U-Net was proposed to obtain accurate prostate location during ultrasound-guided puncture. The ASM-based method was applied for key point selection in the segmentation of prostate ultrasound images from coarse to fine points. The BMGM is easy to plug into any network. The experimental results show that the proposed BSP U-Net has good performance on prostate ultrasound image segmentation in terms of several evaluation indexes.

## Data availability statement

Publicly available datasets were analyzed in this study. This data can be found here: https://challenge.isic-archive.com/data/.

## Author contributions

All authors contributed to the article and approved the submitted version.

## Funding

This work was supported by the National Natural Science Foundation of China (Grant No. 62171125, No. 62141401), the 67th National Postdoctoral Program (Grant No. 2020M671277), the Natural Science Foundation of Jiangsu Province (Grant No. BK20190159), the Science and Technology Project of Changzhou City (Grant No. CE20215045), the Key Laboratory of Computer Network and Information Integration (Southeast University) of the Ministry of Education (Grant No. K93-9-2021-08), and the Key Medical Physics Laboratory of Changzhou (Grant No. CM20193005).

## Conflict of interest

The authors declare that the research was conducted in the absence of any commercial or financial relationships that could be construed as a potential conflict of interest.

## Publisher’s note

All claims expressed in this article are solely those of the authors and do not necessarily represent those of their affiliated organizations, or those of the publisher, the editors and the reviewers. Any product that may be evaluated in this article, or claim that may be made by its manufacturer, is not guaranteed or endorsed by the publisher.
